# Supported Catalysts Useful in Ring-Closing Metathesis, Cross Metathesis, and Ring-Opening Metathesis Polymerization

**DOI:** 10.3390/polym8040140

**Published:** 2016-04-12

**Authors:** Jakkrit Suriboot, Hassan S. Bazzi, David E. Bergbreiter

**Affiliations:** 1 Department of Chemistry, Texas A & M University, College Station, TX 77840, USA; jakkrit.suriboot@chem.tamu.edu; 2 Department of Chemistry, Texas A & M University at Qatar, P.O. Box 23874, Doha, Qatar; bazzi@tamu.edu

**Keywords:** ROMP, supported catalysts, olefin metathesis, green chemistry

## Abstract

Ruthenium and molybdenum catalysts are widely used in synthesis of both small molecules and macromolecules. While major developments have led to new increasingly active catalysts that have high functional group compatibility and stereoselectivity, catalyst/product separation, catalyst recycling, and/or catalyst residue/product separation remain an issue in some applications of these catalysts. This review highlights some of the history of efforts to address these problems, first discussing the problem in the context of reactions like ring-closing metathesis and cross metathesis catalysis used in the synthesis of low molecular weight compounds. It then discusses in more detail progress in dealing with these issues in ring opening metathesis polymerization chemistry. Such approaches depend on a biphasic solid/liquid or liquid separation and can use either always biphasic or sometimes biphasic systems and approaches to this problem using insoluble inorganic supports, insoluble crosslinked polymeric organic supports, soluble polymeric supports, ionic liquids and fluorous phases are discussed.

## 1. Introduction

**Figure 1:**
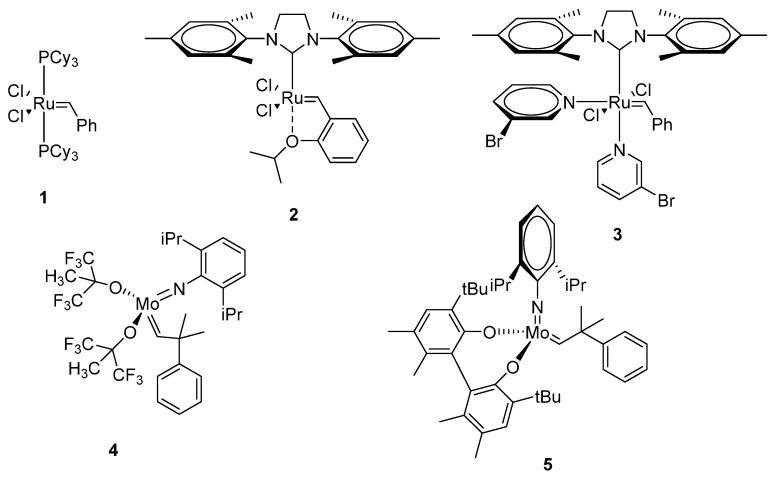
Examples of olefin metathesis catalysts **1**–**5**.

The use of metal complexes in catalysis has become a standard practice in organic synthesis. Olefin metathesis is among these various catalytic reactions. The carbon-carbon double bond rearrangements effected by olefin metathesis have developed into one of the most powerful tools in organic synthesis due to the facility with which this chemistry constructs carbon-carbon double bonds and the compatibility of this reaction with other functionalities [1,2]. The search for novel applications of olefin metathesis reactions and study of improvements of the catalysts’ reactivity, stability, and selectivity have been extremely active fields since the breakthrough introduction of the current two most recognized alkylidene-types of catalyst families of ruthenium and molybdenum based complexes introduced in the 1980s by the Grubbs and Schrock groups, respectively (Figure 1) [3,4]. Examples of these catalysts are bis(tricyclohexylphosphine)benzylidine ruthenium(II) dichloride (a Grubbs 1st generation catalyst) **1** and 2,6-diisopropylphenylimidoneophylidene molybdenum(IV) bis(hexafluoro-*t*-butoxide) (a Schrock catalyst) **4**. A critical aspect of the development of these families of catalysts is the fact that the properties of these olefin metathesis catalysts can be modified by alteration of the organic ligands in these ruthenium and molybdenum complexes. The success and impact of such modifications have been the subject of many studies that have led to a variety of organic ligands for both of the catalyst families (Figure 1). For example, in the case of Ru metathesis catalysts, these studies led to a variety of *N*-heterocyclic carbene complexes including (1,3-bis-(2,4,6-trimethylphenyl)-2-imidazolidinylidene)-dichloro(*o*-isopropoxyphenylmethylene) ruthenium (an example of a Hoveyda-Grubbs 2nd generation catalyst) **2** and [1,3-bis(2,4,6-trimethylphenyl)-2-imidazolidinylidene]dichloro-(phenylmethylene)bis(3-bromopyridine) ruthenium(II) (a Grubbs 3rd generation catalyst) **3**. Similar studies that use ligands to influence catalyst utility have been carried out with Mo catalysts leading, for example, to more useful chiral catalysts like 2,6-diisopropylphenylimido-neophylidene-[(*S*)-(−)-BIPHEN]-molybdenum(IV) catalysts (an (*S*)-Schrock-Hoveyda catalyst) **5**.

Although these studies have led to newer versions of olefin metathesis catalysts that have improved reactivity, air and moisture stability, functional groups tolerance, and stereoselectivity, challenges still remain in using these transition metal complexes. Challenges that remain include: (i) the high cost of the transition metal complexes used as catalysts or precatalysts; (ii) the cost and/or tediousness of the ligand syntheses; and (iii) the potential environmental toxicological or practical concerns that ensue when there is significant metal or ligand contamination in the product. This last issue is a green chemistry issue in that additional process steps have to be used to sequester or remove metals or ligands from products. Such steps increase overall process cost and can be an environmental issue because of the waste that is typically generated in such catalyst/ligand/product separation steps. The last issue is especially critical in pharmaceutical industry and in materials synthesis. In the case of pharmaceuticals, the acceptable level of ruthenium content in products should be less than 10 ppm in the final compound [5,6,7]. An efficient separation of ruthenium impurities is also important in the case of polymeric materials used in electronic or device applications. In addition, other issues can arise. For example, in some syntheses, an efficient separation of ruthenium impurities is important as ruthenium residues can lead to undesirable side-reactions like hydrogenation or alkene isomerization in reaction products [8,9].

There are several ways to approach the three issues noted above. Perhaps the simplest approach is to simply reduce the amount of catalyst that is used in the reaction. Originally, ring-closing metathesis (RCM) reactions required catalyst loadings that were 1–5 mol % or greater depending on specific application. However, this level of catalyst loadings seems to be an overestimate. For example, according to the studies by Mol and Dinger in 2002, they found that catalyst loadings could be much lower in some cases. Their work shows that in suitable cases Ru-catalyzed olefin metathesis can be carried out effectively with catalyst loadings that are several orders of magnitude lower than normally reported [10]. In suitable cases, the effective turnover number for the Ru catalysts can be as high as 600,000. While these studies focused on achieving the maximum turnover number, the reactions with very low catalyst loadings in some cases never reached 100% conversion. Nonetheless, their work demonstrated the potential of lower mol % loadings of Ru catalysts, a potential that has been realized by others such as the Grubbs group who reported success in reducing the catalyst loadings to be as low as 25 ppm in ring-closing metathesis of diethyl diallylmalonate, by employing more sterically demanding and reactive Ru-based catalysts [7].

An alternative strategy to address the issue of separation of transition metal complexes and products in metathesis that has been also used in other transition metal catalyzed chemistry is to design the catalyst and its ligands such that both can be efficiently separated, recovered, and recycled. If such separations recover an active catalyst, a reuse of this catalyst can in effect increase turnover numbers. In any case, a simpler way to separate catalyst residues from products can minimize the effects of catalyst or ligand contamination in products.

Various methods to separate, recover and recycle the catalyst have been developed. The most classical approach is to design a catalyst such that it is coupled to an always insoluble support such as silica gel or a cross-linked and thus insoluble polymer. Such an approach leads to so-called heterogeneized catalysts on supports that are always insoluble before, during and after a reaction. A second approach is to design a catalyst such that it has some sort of phase selective solubility. Examples of this second approach have involved ligands that contain ionic liquid compatible functionality, or fluorocarbon groups that make metathesis catalysts soluble in fluorocarbon solvents, or soluble polymers that lead to catalysts whose solubility mirrors that of the polymer to which they are attached. Unlike cases where catalyst immobilization involves an insoluble support, these catalysts can typically be characterized by conventional spectroscopy. A further advantage of each of these approaches is that the catalysts that can be used as homogeneous catalysts avoiding potential problems of heterogeneous catalysts. However, in each of these three cases, some sort of biphasic separation is required after a reaction to separate the tagged catalyst and ligands from the products. Such separations can involve a simple filtration in the case of heterogeneous supports. Soluble supports usually require some sort of liquid/liquid separation or extraction to separate catalysts and products. The discussion below briefly summarizes some examples where heterogeneous and homogeneous supports are used in metathesis. It then describes in more comprehensive detail examples where phase separable metathesis catalysts are used in ring-opening metathesis polymerization (ROMP).

## 2. Heterogeneous Supported Olefin Metathesis Catalysts

The use of a heterogeneous insoluble support is among the oldest and most widely used tool to effect separation and isolation of catalysts from the products. The first insoluble organic support explored by the scientific community that was later used for homogeneous catalysts was based on a paper that was published by Merrifield—work that subsequently earned Merrifield a Nobel Prize [11]. While Merrifield’s work was directed at using cross-linked polystyrene resins (Merrifield’s resin) in peptide and nucleotide synthesis [11], others recognized that the physical separation of a growing peptide bound to an insoluble organic support he described had potential applications in separation of homogeneous catalysts and products and that if catalysts had stable stationary states that such catalysts could be recycled. Thus, this discovery of Merrifield that focused on peptide synthesis led to many studies using similar insoluble polymeric materials as supports for homogeneous catalysts. As is true in peptide synthesis, catalysts immobilized on these divinylbenzene (DVB)-cross-linked polystyrene supports have the principle advantage of allowing for separation of catalysts and their ligands from a solution of the product in the solvent phase of a reaction mixture via simple filtration. In some cases, heterogeneous supports can also improve catalyst stability and prevent bimolecular decomposition pathways via a phenomenon known as site isolation [12,13,14,15]. However, in other cases, the heterogeneity of an otherwise homogeneous catalyst can change activity in undesirable ways. If the goal of immobilization is to replace a homogeneous catalyst with a separable catalyst with the same activity, this is undesirable.

The discussion below first describes in a general way some examples where olefin metathesis catalysts have been immobilized on always phase separated supports (*i.e.*, insoluble inorganic or cross-linked polymer supports). It then describes similar chemistry that uses sometimes phase-separated supports (*i.e.*, biphasic catalysis or soluble polymers that can be phase separated after a catalytic reaction). Finally, it goes on to discuss in more detail issues associated with the specific example of immobilized ring-opening metathesis polymerization (ROMP) catalysis.

**Figure 2:**
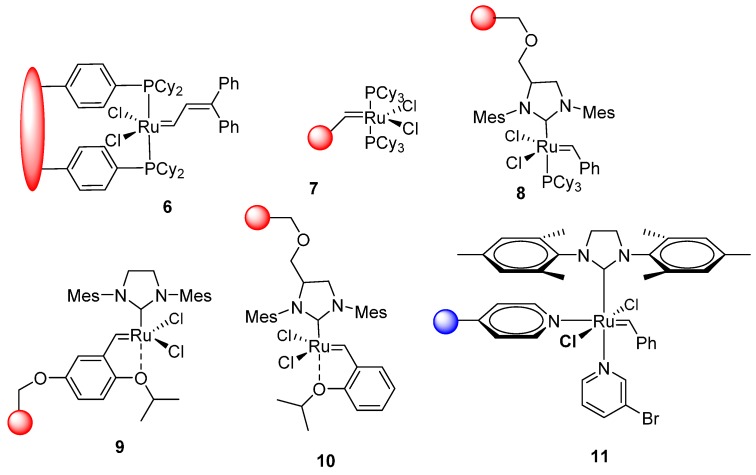
Examples of DVB-PS-supported (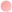 = divinylbenzene cross-linked polystyrene or 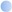 = divinylbenzene cross-linked 4-vinylpyridine) olefin metathesis catalysts **6–11**.

Ru metathesis catalysts like those shown in Figure 2 have been immobilized on divinylbenzene cross-linked polystyrene (DVB-PS). An early example of such catalyst was **6** which was first reported by Grubbs and Nguyen in 1995 [16]. In this example, they used an established type of supported ligand—a phosphine modified DVB-PS. This supported catalyst had extended lifetimes. This was ascribed to the reduced diffusivity of the catalyst molecules on the polystyrene support, which prevents a decomposition pathway that occurs via a bimolecular reaction. However, while **6** was recycled three times in a metathesis reaction of *cis*-2-pentene to form cross metathesis products of 3-hexene and 2-butene, the catalyst lost 20% of its activity after each cycle. This catalyst also had a modest activity that was ascribed to (i) incomplete substitution of phosphine; (ii) the diffusion limit of olefin into the cavities of cross-linked DVB-PS support; and (iii) the local high concentration of phosphine on the support. The amount of Ru leaching in the products was not analyzed. However, given that the Ru complex is immobilized by a labile phosphine ligand, given that the Ru and phosphine ligand dissociate in the reaction, and that the recovered polymer-bound catalyst loses significant activity cycle to cycle, it is likely that there is a significant amount of Ru leaching. Several years later, Barrett and co-workers described an alternative scheme that also used DVB-PS as a support. In this case, they immobilized a Ru pre-catalyst onto DVB-PS using a benzylidine group. In this second approach, Barrett introduced a new concept called the “boomerang” effect [17]. This was a quite different scheme for use of a heterogeneous-supported catalyst. In this case a pre-catalyst 7 was synthesized by a metathesis reaction, shaking Grubbs 1st generation catalyst and a vinyl-substituted DVB-PS derivative for 2 h in dichloromethane. The resulting benzylidene-immobilized pre-catalyst **7** is immobilized on the insoluble resin by a strong bond and the immobilized pre-catalyst was isolated by filtration. According to their report, **7** was indefinitely stable under normal atmospheric conditions with no loss of activity. In the application of **7**, a so-called boomerang reaction was involved. In this scheme, the Ru initially undergoes a metathesis reaction with an alkene of a ring-closing metathesis (RCM) substrate like diethyl diallylmalonate. The resulting metallocycle then forms a new Ru alkylidene and the original vinyl-substituted DVB-PS. The alkylidene complex goes into solution, becoming a homogeneous catalyst effecting ring-closing metathesis (RCM) on the rest of the substrate. Then, when the substrate is consumed, the authors postulate that the remaining soluble alkylidene reacts with the vinyl-substituted DVB-PS. This leads to a recapture of the Ru complex by the resin by a strong bond after the completion of the reaction. This behavior of the ruthenium catalyst where it comes off the resin and returns later was likened to the action of a “boomerang”. In this work, the catalyst was recycled up to three times in an RCM reaction of diethyl diallylmalonate, with addition of 3,3-dimethyl-1-butene (9 mol % based on diethyl diallylmalonate), with Ru contamination in the product of 500 ppm. Catalyst recycling was accomplished by simple filtration of the solid-supported catalyst and the product was isolated from the filtrate by evaporation of the solvent.

Following Grubbs’ and Barrett’s work on polymer-supported catalysts and the other studies of homogeneous Ru metathesis catalysts that had shown the utility of Ru-based alkylidene catalysts that contained *N*-heterocyclic carbene (NHC) ligands, Blechert and co-workers described the synthesis of Grubbs 2nd generation catalysts like **8** that were immobilized on DVB-PS via an *N*-heterocyclic carbene ligand [18]. An advantage of this approach using *N*-heterocyclic carbene (NHC) ligands to immobilize a Ru complex is that an NHC ligand is a stronger sigma donor than phosphine ligands. Unlike phosphine ligands like those in **7** that are thought to dissociate from the ruthenium center to initiate catalysis [19], the NHC ligand should remain bound to the ruthenium center before, during, and after the metathesis reaction. Thus, leaching of Ru from the resin was expected to be reduced [18]. In the first example of this chemistry, the solid-supported catalyst **8** was prepared by ligand exchange between a DVB-PS-supported *N*-heterocyclic carbene ligand and the phosphine ligand on Grubbs 1st generation catalyst **1**. The resulting DVB-PS supported heterogeneous ruthenium catalyst was characterized by ^31^P NMR and IR spectroscopy. The general reactivity of **8** was demonstrated by the use of **8** in various metathesis reactions including RCM and yne-ene metathesis reactions. In the case of ring-closing metathesis reaction of diethyl diallylmalonate, up to four cycles of cyclization of diethyl diallylmalonate to 4,4-dicarboethoxycyclopentene could be effected using **8**. These reports stated that the products were obtained as colorless solids or oils. While this visual assay of Ru leaching is promising, a more quantitative assay of the amount of Ru leaching was not reported. This work was subsequently extended in a report where the Blechert group reported solid-supported Ru metathesis catalysts like **9** and **10** that had high stability, activity, and recyclability [20]. In recycling experiments using these two catalysts, both catalysts were recycled up to four times in RCM reaction of diallyltosylamide. However, the ability to catalyze cross metathesis (CM) reactions of complex **9** was showed to be higher than that of complex **10**. For example, the conversion of CM between acrylonitrile and 4-pentenyl benzoate catalyzed by **9** was 98% in 12 h while the conversion was only 15% in 12 h in the case of **10**. The authors suggested that the superior activity of complex **9** owe to the ability of the catalyst to dissociate in solution, becoming homogeneous active species, unlike complex **10**. The leaching of Ru in the products was not described.

The invention of new highly active ruthenium olefin metathesis catalyst pyridine-ligated Grubbs 3rd generation catalysts has led to a new type of Ru catalyst which can catalyze cross metathesis reactions of a broader range of substrates and afford polymers with narrow polydispersity (PDI) by a ring-opening metathesis polymerization (ROMP). Grela and Kirschning reported the first example of a solid-supported version of this catalyst in 2005 [21]. Their report noted the possibility of using this supported catalyst in a continuous flow process due to the ability to reload the catalytic species onto the same solid support. Thus, even if leaching were to occur, the catalyst could be easily be regenerated. The recyclability of **11** was tested in a ring-closing metathesis reaction of diethyl diallylmalonate at 110 °C. This solid-supported catalyst showed activity up to 5 cycles but a decrease in product yield was noted in each subsequent cycle. The authors ascribed this to the thermal instability of **11** or leaching of Ru from the weakly bound pyridine ligands. However, the authors were able to show that **11** could indeed be reactivated by washing/Ru re-addition protocol (1 M HCl, 1 M NaOH, H_2_O, MeOH, toluene, then addition of **3**). Ru contamination in the products was not mentioned.

**Figure 3:**
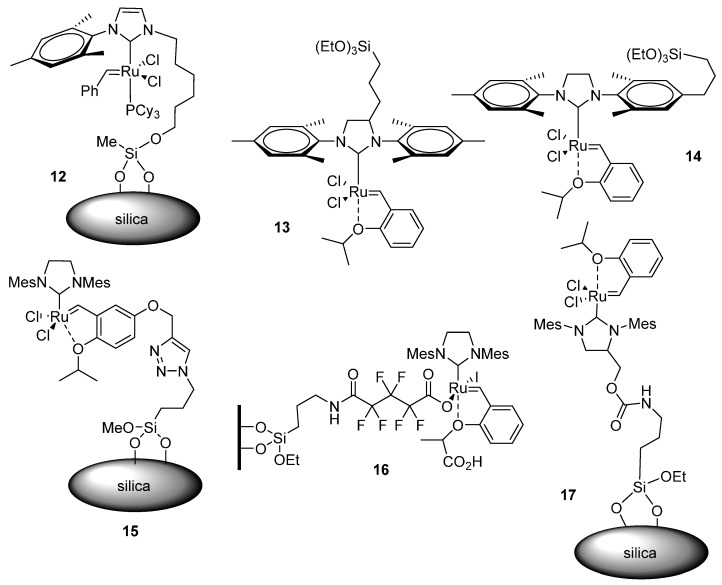
Examples of silica-supported olefin metathesis catalysts **12**–**17**.

The other general strategy for catalyst immobilization on insoluble supports commonly is to use inorganic supports. Silica-based materials are the most common examples of these sorts of solid supports. Select examples of Ru metathesis catalysts immobilized on silica are shown in Figure 3. Fürstner and co-workers reported the synthesis of an immobilized Grubbs 2nd generation ruthenium complex on silica gel using hydroxyalkyl groups on *N*-heterocyclic carbene ligand 12 [22]. This immobilized catalyst was successfully recycled in RCM reaction of diethyl diallylmalonate through three cycles. The catalyst **12** was separated from the RCM product by simple filtration. The crude product was isolated as colorless liquid. The authors did report a detailed analysis of Ru leaching noting that the products had *ca.* 250 ppm of Ru contamination based on ICP-MS analysis. Grubbs and co-workers have also described other versions of silica-supported olefin metathesis ruthenium catalysts **13** and **14** [23]. The silica-supported complexes **13** and **14** were competent catalysts in RCM and CM reactions with reactivity that was comparable to that of their homogeneous analogs. The catalytic activity toward an RCM reaction of diethyl diallylmalonate of catalyst **14** (31% conversion after 10 min) was shown to have slightly lower than the catalytic activity of **13** (67% conversion after 10 min). The authors also showed that immobilized ruthenium catalyst **13** can be recycled up to eight times in RCM reaction of allyl(2-methyl-2-propenyl)malonate with conversions of substrate to product in the 60%–80% range when reaction time was 2 h, conversions that could reach 100% with reaction times of 12 h. The authors suggested that these catalysts improve recyclability and eliminate issues associated with the decomposition of the ruthenium complex via bimolecular pathway. Such immobilized ruthenium catalysts on silica support have less intermolecular activity between the catalysts—the same phenomena reported earlier by Grubbs’ group for site isolated DVB-PS supported species [16]. The analysis of the Ru leaching in this case would seem to support this claim. In this example, the ruthenium leaching studied by ICP-MS revealed the contamination level in products to be less than 5 ppb for those prepared by both **13** and **14**. This is especially notable since it is by several orders of magnitude the lowest Ru leaching ever reported. Ying group also described using click chemistry for the immobilization of Hoveyda-Grubbs type complexes on nanoporous silica **15** [24]. The catalyst they prepared exhibits good activity and stability as well as recyclability. In addition, these authors demonstrated that this catalyst can be used in a circulating flow reactor. The catalyst was reused in RCM reaction of diethyl diallylmalonate over 8 times with overall conversions of 90% with Ru leaching levels of 11.3 ppm at the first 60 min and 1.6 ppm at 180 min based on ICP-MS analysis of the isolated products. Yet another example of silica-bound Ru metathesis catalysts was reported by Balcar and co-workers who used commercially available molecular sieves as supports for the Ru catalyst **16** [25]. These SBA-15 molecular sieves possess several advantages including high surface area, narrow pore size distribution, and high thermal and mechanical stability. The solid-supported ruthenium complex **16** on such sieves was shown to be competent as an RCM catalyst using diethyl diallylmalonate as a substrate. The leaching of Ru into RCM product was found to be as low as 17 ppm. However, attempts to recycle this catalyst were unsuccessful, with the conversion reaching 90% for only two cycles. More recently, Monge-Marcet and co-workers also reported a synthesis of recyclable silica-supported Hoveyda-Grubbs type complex using an NHC ligand **17** [26]. This catalyst proved to be recyclable for RCM reaction of diethyl diallylmalonate with Ru contamination of 221 ppm found in the product based on ICP-MS of products from the first cycle. Ru leaching in subsequent cycles was not measured but would likely be less as Ru leaching in the first or second cycles of a catalytic reaction could reflect undetectable issues in the synthesis of a catalyst. If a catalyst were only 99% pure (pure by most analyses used in synthesis), the 1% impurity would represent most or all of the observed Ru leaching but would not be relevant in later cycles.

**Figure 4:**
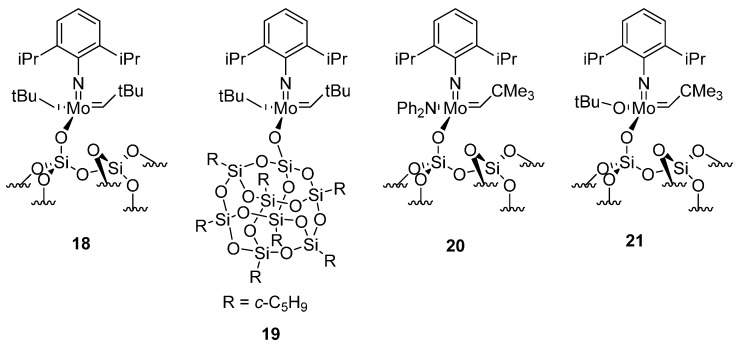
Examples of silica-supported olefin metathesis catalysts **18**–**21**.

Schrock’s molybdenum olefin metathesis catalysts have also been supported on always insoluble supports. Some examples of such silica-supported catalysts supports are shown in Figure 4. For example, Schrock and co-workers described the synthesis of well-defined surface immobilized catalysts **18** and **19** [27]. These two silica-supported catalysts showed very similar activities in cross metathesis reaction of ethyl oleate (EO) with TOF (turnover frequency measured after 5 min of reaction expressed in mol of substrate converted per mol of Mo per sec) of 0.04 and 0.03, respectively. However, the silica-supported **18** was reportedly more stable than **19**, a difference that was ascribed to the site isolation of metal complexes on the silica support [28]. The stability of **18** and **19** were determined by the time needed to reach equilibrium in the CM reaction of ethyl oleate (*i.e.*, around 50% conversion), which was 1 and 24 h, respectively (the longer the time was presumed to reflect a faster decomposition rate for the catalyst). Shortly later, Schrock and co-workers developed more active, stable, and selective silica supported molybdenum olefin metathesis catalyst **20** [29]. The increase in reactivity was achieved by replacing one imido group with a siloxy group from the surface. Keeping one remaining imido ligand enhanced the stability of the molybdenum catalyst. This catalyst was also tested its activity in CM of ethyl oleate. The results showed that the TOF of **20** was 0.15 and the time needed to reach reaction equilibrium was only 10 min. More recently, Schrock group described the silica immobilized molybdenum alkene metathesis catalyst **21** that showed enhancement in metathesis activity compared to its low molecular weight analog in CM reactions of ethyl oleate. The TOFs of **21** and its low molecular weight analog were 0.07 and 0.01, respectively [30]. None of these reports reported an analysis of Mo leaching.

**Scheme 1:**
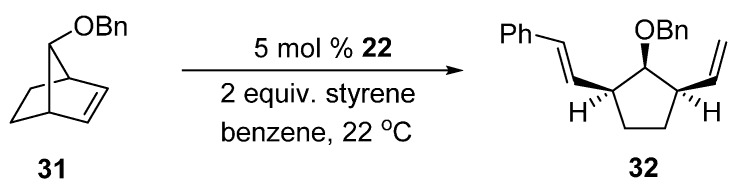
Asymmetric Ring-Opening Cross Metathesis Reaction of **31** catalyzed by **22**.

**Figure 5:**
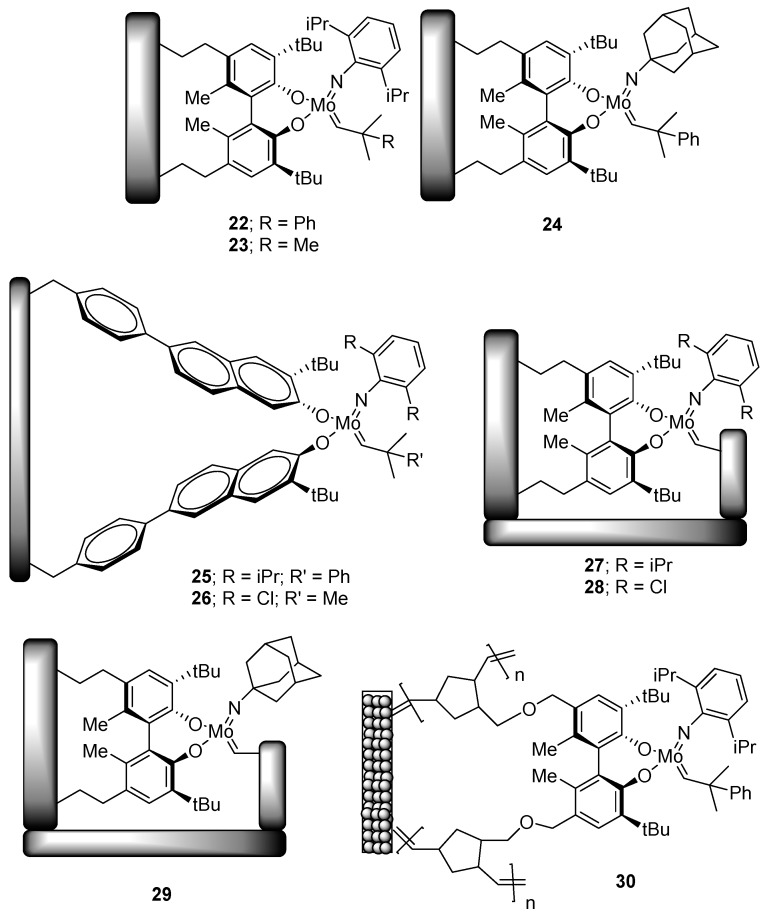
Polystyrene-based-supported Schrock catalysts **22**–**26** and polynorbornene-based-supported Schrock catalysts **27**–**30**.

The first recyclable supported chiral olefin metathesis catalyst was reported by Hoveyda and Schrock in 2002 [31]. Examples of these catalysts are shown in Figure 5. The polystyrene-supported catalyst **22** showed similar activity to its homogeneous analog both in terms of yield and enantioselectivity in asymmetric ring-opening cross metathesis reaction of (1*R*,4*S*,7*R*)-7-(benzyloxy)bicyclo[2.2.1]hept-2-ene **31** and styrene to yield product **32** (92% yield and 98% enantiomeric excess (ee)) (Scheme 1). The polymer-supported complex can be recycled three times but conversion significantly dropped in the third cycle. However, there was little difference in enantioselectivity in the product in the three cycles. This catalyst also affords good recoverability of the catalyst. The reported leaching of molybdenum in the product was 3% of the charged catalyst. Three years later Hoveyda and Schrock described new immobilized catalysts using both polystyrene- and polynorbornene-based supports, **23**–**26** and **27**–**29**, respectively [32]. These polymer-supported catalysts can be used in asymmetric ring-opening cross metathesis reaction of the same substrates mentioned earlier and the catalysts can be separated from the reaction mixture by simple filtration. The leaching of molybdenum in the product was found to be as low as 1%, with reactivity and enantioselectivity similar to their homogeneous counterparts. The synthesis of a polynorbornene monolith-supported Schrock-type catalyst **30** was also reported by the groups of Buchmeiser and Fürstner [33]. The monolith-supported chiral catalyst **30** was used with excellent product yields and with excellent results in term of recovery of the catalyst in asymmetric RCM reactions of 3-(allyloxy)-2,4-dimethylpenta-1,4-diene and its derivatives. In most cases, the reaction proceeded with yields that exceeded 99% with the molybdenum contamination in the products being less than 2% in all cases. The enantioselectivity was also comparable to its homogeneous analog with slightly lower enantiomeric excesses.

## 3. Homogeneous Supported Olefin Metathesis Catalysts

Up to this point, this discussion has focused on the immobilization of olefin metathesis catalysts on heterogeneous supports. While this has been a common technique to isolate and recycle the catalysts, it is not the only possible scheme for catalyst product separation and catalyst recycling. Soluble phase tag methods used in combinatorial chemistry and in peptide synthesis have also been developed as an alternative tool for separation process between catalysts and the products. In these cases, the strategy is to use two different phases to recover catalysts. The presence of the two phases can result from perturbation of a homogeneous reaction mixture or can involve two liquid phases. Thus, the supports for this technique do not always have to be macromolecules—small molecules like fluorous tags or ionic tags can also be used to effect catalyst/product separation and catalyst recycling. Such soluble supports have potential advantages in that they can avoid some of the problems of heterogeneized catalysts that include more complicated analyses and the observation that the reactivity and selectivity of an immobilized catalyst differ from what is seen with an optimized homogeneous catalyst analog. These problems reflect the fact that the advantage of heterogeneous catalysts, which is their ease of separation from the reaction mixture, is an issue not just at the separation step after the reaction but also during the reaction and during the catalyst synthesis. The strategy in which phase tag that is soluble is used can avoid this issue in that catalyst soluble supports or phase tags allow the catalysts to be synthesized and characterized in homogeneous solution and allow them to carry out their reaction as homogenous catalysts. Such tags are only used to effect separation after the reaction is complete.

**Figure 6:**
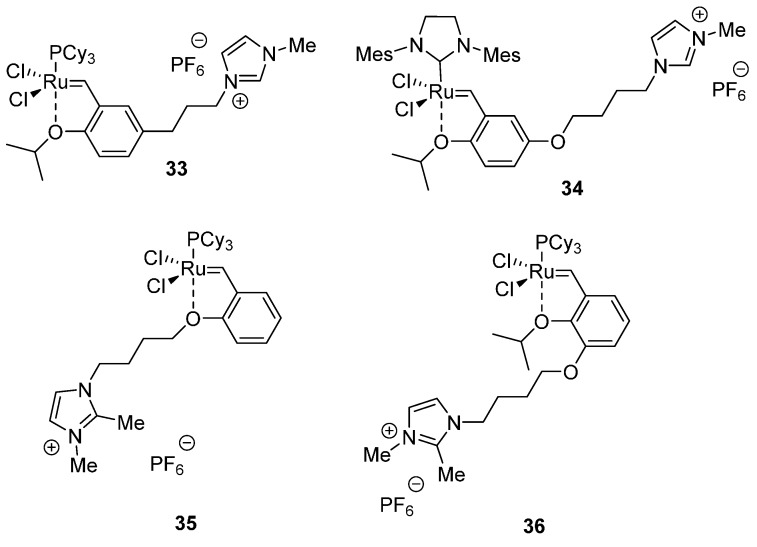
Ionic liquid-tagged ruthenium catalysts **33**–**36**.

An example of this approach is the use of ionic liquid (IL) immobilized catalysts (Figure 6). Ionic liquids are alternative solvents that are useful because of their unique properties including non-volatility, high stability, and good recyclability [34]. These alternative solvents are immiscible with most organic solvents. Thus, they can be used in catalytic reactions as a recyclable phase. Buijsman and co-workers reported the use of Grubbs 1st generation catalyst **1** and Grubbs 2nd generation catalyst in an RCM reaction of 1,5-diallyl-3-benzyl-5-isobutylimidazolidine-2,4-dione in 1-butyl-3-methylimidazolium hexafluorophosphate (BMI PF_6_) as an ionic liquid solvent. These catalysts were recycled up to three times with Ru residues of *ca*. 5000 and 1600 ppm in the product, respectively [35]. One year later, Dixneuf reported the use of BMI PF_6_ as solvent in an RCM reaction of diallyltosylamide catalyzed by a ruthenium benzylidine salt and was able to recycle the catalyst for two cycles [36]. Following this work, Guillemin and co-workers introduced the ionic liquid-tagged ruthenium catalyst **33** which was synthesized in order to minimize the leaching of the catalyst from the ionic liquid phase [37]. This ionic liquid-bound catalyst **33** completed an RCM reaction of diallyltosylamide with BMI^.^PF6 as a solvent at 60 °C in 45 min using 2.5 mol % of **33**. The isolation of the product was achieved by extraction with toluene and the ionic liquid phase containing **33** was reused for an RCM reaction of diallyltosylamide for 8 cycles. Importantly, this catalyst was stable enough to catalyze the ninth cycle of this RCM reaction without any loss in activity after three months. Yao and Sheets reported the synthesis of a similarly stable ionic liquid-tagged catalyst **34** [38]. This catalyst too was recycled very effectively through 17 cycles in the RCM reaction of diallyltosylamide without any loss in activity. In contrast, a similar untagged analog of **34** lost its activity in the second and subsequent runs. In 2007, Dixneuf and co-workers reported the synthesis of **35** and **36**, an improved version of **34** [39]. However, while the activities of both catalysts, toward the same substrate mentioned earlier, were good for the first cycle, the catalyst activities significantly dropped in the second cycle. In general, these studies relied on measuring catalyst reactivity as a basis for catalyst recyclability. The amount of Ru leaching was typically not mentioned except in a few cases.

**Figure 7:**
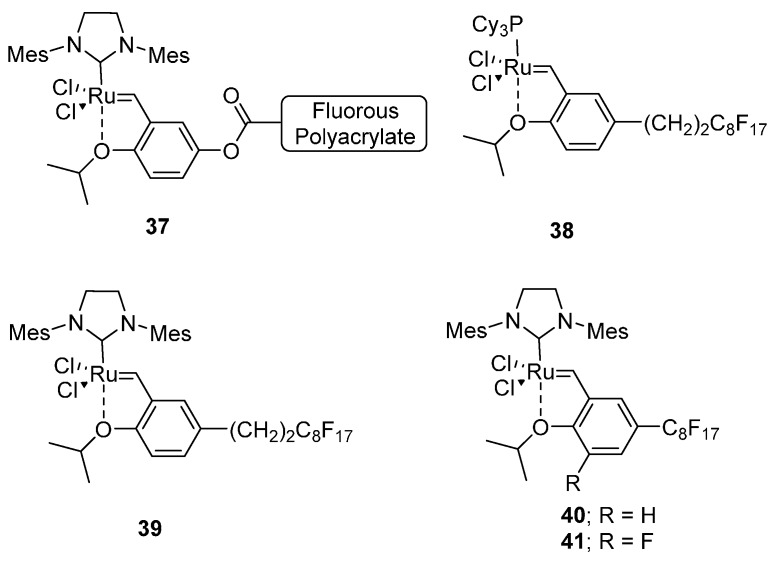
Fluorous-tagged ruthenium catalysts **37**–**41**.

An alternative scheme for liquid/liquid separation that has been used in metathesis and in other chemistry is fluorous phase technology. In this scheme, catalysts are modified with fluorinated phase tags to facilitate separation, recovery and recycling in perfluorinated solvents [40]. Examples of fluorous-tagged Ru catalysts are shown in Figure 7. Since the first report by Horváth [41], fluorous biphasic catalysis has greatly expanded and developed into a general strategy for separations [42]. This chemistry typically uses a mixture of organic and fluorous solvents, relying on the fact that fluorous solvents are often immiscible with most organic solvents at room temperature and such solvent mixtures are often biphasic. Such solvent mixtures can be used in two ways. In one type of experiment, a reaction is carried out under biphasic conditions with a biphasic liquid/liquid separation after a reaction. Alternatively, in some cases fluorous and organic solvents become miscible at elevated temperature. In those thermomorphic systems, a reaction can be effected under monophasic conditions and the catalyst and the fluorous phase can be separated from an organic product in an organic phase at room temperature when the biphasic mixture reforms and the phases can be separated one from another by a gravity-based liquid/liquid biphasic separation. In 2004, Yao and Zhang reported the immobilization of a Grubbs-type catalyst on poly(fluoroalkyl acrylate) **37** [42]. The catalyst **37** was used in RCM reactions of diallyltosylamide in a monophasic PhCF_3_/CH_2_Cl_2_ (1:19 *v*/*v*) solvent system. Extraction of the fluorous species using perfluorohexane (FC-72) and EtOAc after each reaction allowed the catalyst to be recovered and reused. The authors were able to recycle this fluorous-tagged ruthenium catalyst for 20 cycles. However, leaching was not described. Inspired by this work, Curran group reported the study of other recoverable metathesis catalysts using lighter fluorous supports, with only 17 fluorine atoms per Ru in **38** and **39** *versus* 170 fluorine atoms per Ru in **37** [43]. These catalysts **38** and **39** show similar activity to their non-fluorous-bound analogs in RCM reaction of diallyltosylamide. However, the separations of catalysts **38** and **39** involved the use of fluorous silica gel rather than a liquid/liquid extraction. Thus, extra solvents including acetonitrile were needed to obtain the product and ether was needed to recover the fluorous-tagged catalyst. The recovered catalyst can be reused for at least five cycles with the average product yield of 97%. Later on, Matsugi and co-workers reported two other light fluorous-tagged catalysts **40** and **41** [44]. Compared to **39**, fluorous-tagged catalyst **40** had an improvement in activity with similar recyclability (90% recovery of catalyst), while **41** showed higher activity than both **39** and **40** but was not recoverable [44]. The fluorous-tagged catalyst **40** was recycled and reused in an RCM reaction of diethyl diallylmalonate for five times. The product yield was 95%–100% in each cycle.

**Figure 8:**
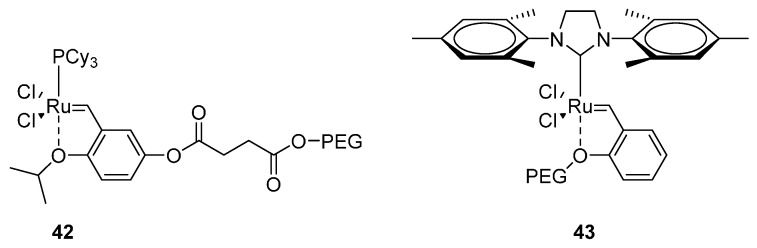
PEG-supported Hoveyda-Grubbs catalyst **42** and **43**.

Soluble polymeric supports are an alternative to ionic or fluorous tags. Poly(ethylene glycol) (PEG) is one of the most widely used such supports for reagents and organometallic complexes [45,46,47]. PEG has been used both to bind catalysts and has been used as a solvent [48]. Examples of PEG-bound Ru metathesis catalysts are shown in Figure 8. Although PEG and PEG-bound catalysts are soluble in many organic solvents including water, they are insoluble in solvents like hexane, diethyl ether, and cold ethanol. Thus, these PEG derivatives can be utilized to separate catalysts and products for catalyst recovery and recycling by either solvent precipitation or liquid/liquid extraction. An early example of the synthesis and application of a PEG_5000_-bound ruthenium metathesis catalyst **42** was reported by Yao in 2000 [49]. This soluble polymer-supported catalyst **42** was recycled 8 times in the RCM reaction of allyl(4-pentenyl)tosylamide with more than 92% conversion in each cycle. The catalyst was recovered by precipitation with diethyl ether. Although the author reported the success in catalyst recycling, the analysis of Ru leaching was not reported. It also should be noted that this type of process for catalyst recovery requires a large excess of the ether solvent—a possible issue in green chemistry terms. In 2003, Lamaty and co-workers described the study of a PEG_3400_-bound Hoveyda-Grubbs 2nd generation catalyst **43** [48]. The soluble support was attached to the benzylidene ligand *ortho* to the metal carbene. This catalyst can catalyze RCM reaction of diallyltosylamide for 5 cycles at room temperature. The recovery of this catalyst from RCM reaction was also carried out by precipitation in diethyl ether and filtration. In this work, the authors carried out some additional characterization of the recovered PEG-bound Ru complex. The precipitate was analyzed by ^1^H NMR spectroscopy, measuring the intensity of the vinyl proton of the alkylidene group in the catalyst at 16.50 δ. The results showed that only 57% of **43** was recovered after the first cycle of RCM reaction.

**Figure 9:**
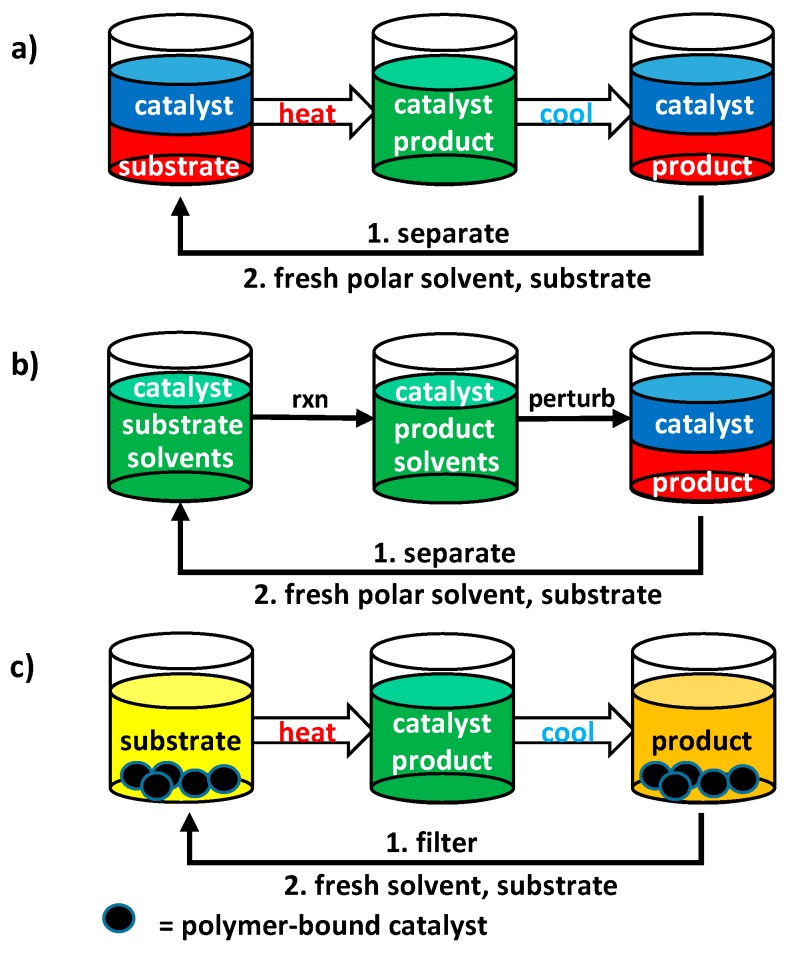
Schematic representation of (**a**) thermomorphic liquid/liquid separation where a biphasic mixture of **polar** and **nonpolar** solvent is heated to form a **miscible solvent mixture**; (**b**) a latent biphasic liquid/liquid separation where a room temperature mixture of **miscible solvents** is used to effect a reaction and the solvent mixture is perturbed (e.g., by addition of another solvent (e.g., water) or some salt) to form a separable biphasic mixture of **polar** and **nonpolar** solvents; and (**c**) a thermomorphic solid/liquid separation system where a mixture of substrate and **insoluble polymer-bound catalyst** is heated to form a **monophasic solution** that is in turn cooled to form a **precipitate of the polymer bound catalyst** that is then separated from the **product solution** by filtration.

**Figure 10:**
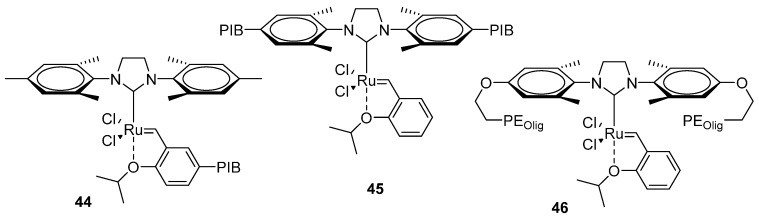
Polyolefin-supported Hoveyda-Grubbs catalysts **44**–**46**.

The Bergbreiter and Bazzi groups have also described the use soluble polymer supports for ruthenium olefin metathesis catalysts. In their work, they focused on soluble polyolefin oligomers that can be more efficiently separated as an alternative to PEG supports whose separation typically generates large volumes of solvents waste during the polymer precipitation step. These alternative polymers are polyethylene (PE_Olig_) and polyisobutylene oligomers (PIB). Several techniques for separation of these types of polymer-supported species including liquid/liquid separation and solid/liquid separation shown in Figure 9 can be used. The first synthesis and application of PIB-supported Hoveyda-Grubbs type catalyst using such polymers was described in 2007 [50]. In this case, the PIB (*M*_n_ = 1000) was attached to the catalyst as a benzylidene ligand following a prior strategy described by Barrett [17]. This pre-catalyst **44** was then used to catalyze RCM reactions in heptane. The products were then separated from the catalyst solution by an acetonitrile extraction. In some cases, the RCM product self-separated from the catalyst. In such cases where the product precipitated from the heptane solution, a filtration was only needed to isolate the product. This PIB-supported ruthenium complex **44** was reused for at least five cycles. The Ru leaching into the product was measured and varied from 20 to 1000 ppm, suggesting that the “boomerang” scheme for recycling the catalyst was not as effective as desired. In order to improve the recoverability of the PIB-supported ruthenium catalyst, an alternative type of catalyst was prepared where the PIB (*M*_n_ = 1000) chains were attached to the non-dissociating *N*-heterocyclic carbene ligands of a Hoveyda-Grubbs catalyst as in complex **45** [51]. This catalyst design led to improvements in both the consistency and effectiveness of Ru catalyst/product separation. Leaching levels dropped to values as low as 73 ppm (0.37% of the charged Ru catalyst). Moreover, catalyst **45** could be reused for 20 cycles. More recent work from our group on polymer-supported olefin metathesis catalyst explored the use of Polyethylene oligomer (PE_Olig_) (*M*_n_ = 550) as catalyst supports [52]. The unique property of PE_Olig_ is that it is like higher molecular weight polyethylene and does not dissolve in any solvent at room temperature. However, like high molecular weight polyethylene, it does dissolve at elevated temperature. Moreover, polyethylene oligomers that are commercially available as 500–2000 Da materials are soluble in toluene or THF at relatively modest temperatures like 65 °C. A PE_Olig_-supported Hoveyda-Grubbs catalyst **46** formed from PE_Olig_ with an *M*_n_ of 550 Da can be dissolved in a solvent with modest heating to form a monophasic solution. The complex **46** quantitatively precipitates on cooling. Thus, **46** can be added to substrate, the suspension that forms can be heated to form a monophasic reaction solution, and the catalyst can be separated from a solution of the product by simply cooling it back to room temperature. Filtration then effects phase separation between the solid catalyst species and the product solution. In reports describing this catalyst, the PE_Olig_-supported catalyst **46** was used in a variety of RCM reactions for at least eight cycles with the Ru leaching based on ICP-MS analysis that was less than 0.3% based on the amount of charged Ru. Examples of these polyolefin-supported Ru metathesis catalysts are shown in Figure 10. The effectiveness of the catalyst/product separation in these processes was further tested in sequential reactions where the same sample of **46** was used to carry out three successive RCM reactions. This experiment not only showed that Ru leaching was low but that the product of one reaction was not detectable in a subsequent cycle that used the same catalyst used in a prior different reaction.

Up to this point, this review has focused on background examples of ring closing and cross metathesis reactions where metathesis catalysts are separated either by liquid/solid or liquid/liquid separations. In these applications, the need for a separation strategy depends on the reactivity of the Ru catalyst. Catalysts with TONs that approach 10^5^ or 10^6^ may not even need a support and a separation strategy. This however is not true in polymerization reactions where no chain transfer occurs. In such cases, the living character of a ring-opening metathesis polymerization in a polymerization leads to one metal center being used for each polymer molecule synthesized. In these cases metal or metal-ligand separations are more critical. The balance of this review will discuss metathesis catalysts and ligand separation from products in this more demanding context.

## 4. Supported Catalysts in Ring-opening Metathesis Polymerization (ROMP)

**Scheme 2:**
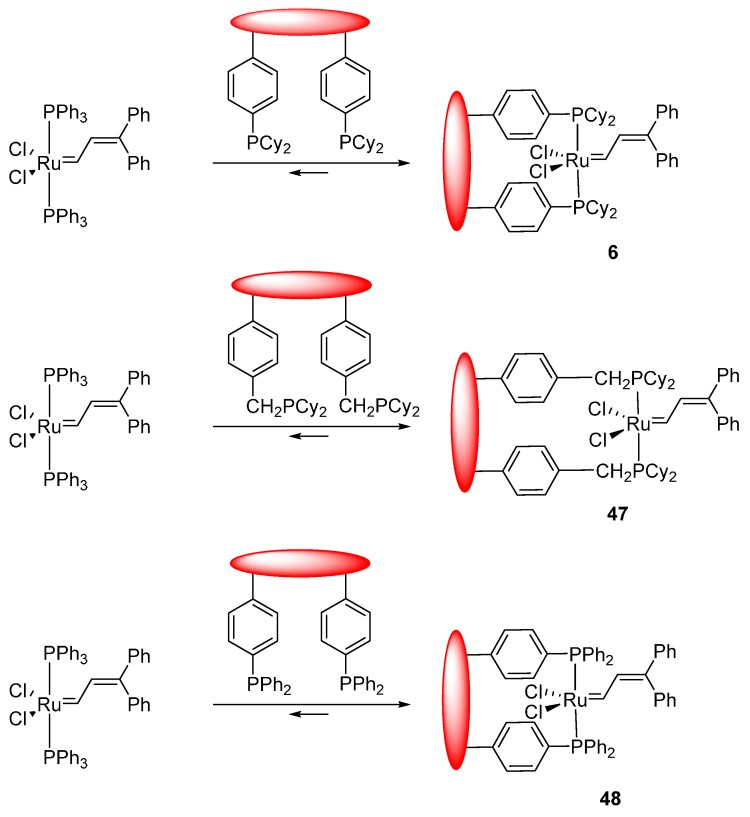
DVB-PS-Supported Olefin Metathesis Catalysts **6**, **47**, and **48**.

The first example of supported catalyst used in ROMP is also the first well-defined polymer-supported olefin metathesis catalyst [16]. In this study by Grubbs and Nguyen, a series of polystyrene-divinylbenzene (DVB-PS)-supported Cl_2_(PR_3_)_2_Ru=CH-CH=CPh_2_ olefin metathesis catalysts **6**, **47**, and **48** were synthesized as shown in Scheme 2. These solid-supported catalysts showed activities that were similar to that seen for their homogeneous analogs where the catalyst reactivity varied depending on the nature of PS-supported phosphine ligands. For example, while catalyst **48** can only catalyze ROMP reaction of highly strained cyclic olefins such as norbornene, **6** and **47** can catalyze ROMP of the less strained cyclic olefins such as cyclooctene. Although **6**, **47**, and **48** were competent catalysts for ROMP of norbornene, the PDIs of the resulting polymer products were significantly higher than those prepared by the homogeneous analogs. For example, polynorbornene prepared from **6** had PDI values of 5.5, while **1** could be used to prepare similar polymers with PDI values ranging from 1.1 to 1.3. The broader molecular weight range for the polynorbornene prepared by the immobilized catalyst was postulated to be a result of the slow initiation rate of immobilized catalyst that could reflect its inhomogeneity or perhaps the fact that the actual catalyst is a complex that dissociates from the support. The amount of Ru contamination in the ROMP products was not reported.

**Scheme 3:**
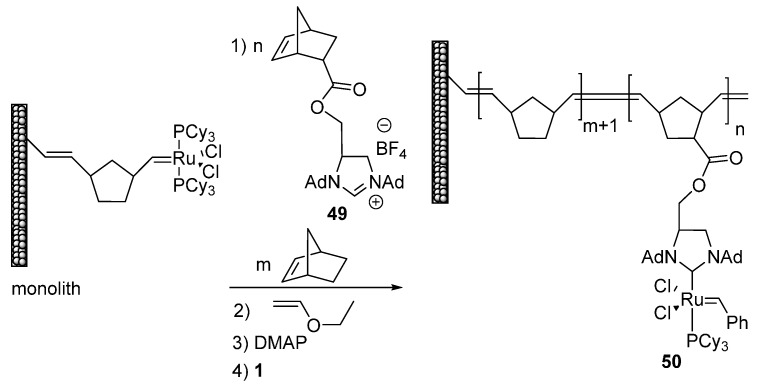
Synthesis of Monolith-Supported Ruthenium Olefin Metathesis Catalyst **50**.

Alternative insoluble organic supports that have been used as catalyst supports for Ru ROMP catalysts include monolithic materials. While such materials have been known since the 1970s [53] but have received more attention [54,55] after studies by Fréchet and Svec highlighted their utility as high-performance separation media, scavengers, and reagent supports [56,57]. Such media have been used too as metathesis catalyst supports as noted above. That work includes studies by Buchmeiser whose group described a synthetic route to a monolith-supported ruthenium olefin metathesis catalyst **50** (Scheme 3) [58]. The monolith was generated through ring-opening metathesis copolymerization of norbornene (NBE) and 1,4,4a,5,8,8a-hexahydro-1,4,5,8-*exo*-*endo*-dimethanonaphthalene (DMN^.^H6) in the presence of dichloromethane and 2-propanol within a borosilicate column. The functionalization of the catalyst onto the monolith was achieved by grafting a mixture of compound **49** and norbornene onto this solid support. After the grafting process was complete, it was terminated by addition of ethyl vinyl ether. The resulting grafts contained imidazolium salts that were then deprotonated with dimethylaminopyridine (DMAP) to generate an NHC ligand. The immobilization of a ruthenium catalyst onto the monolith support using these NHC ligands was then achieved by treating this monolith-immobilized NHC ligand with **1**. As noted above, the monolith-supported ruthenium complex **50** showed high activity toward RCM. This RCM catalyst was also used in ROMP reactions, where the *cis* and *trans* ratio of ROMP products of norbornene and *cis*-cyclococtene were the same as those obtained using the analogous homogeneous catalyst. In the ROMP chemistry, the PDI of polymer products ranged from 1.2 to 2.6. However, while the extent of Ru leaching in RCM products was reported as 70 ppm, Ru leaching in the ROMP products was not reported.

**Scheme 4:**
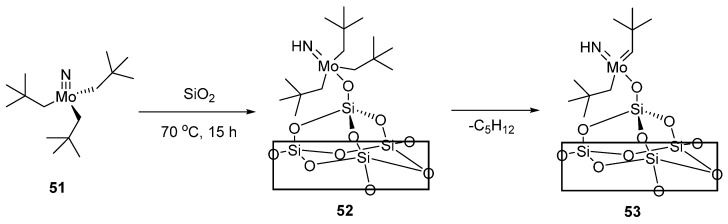
Synthesis of Silica-Supported Schrock Catalyst **53**.

Schrock’s molybdenum catalysts that are used in ROMP have been also immobilized on heterogeneous supports. However, perhaps because of their sensitivity toward air and moisture, there are relatively few reports discussed about immobilizing these Mo-based ROMP catalysts onto solid supports. One example was reported by Basset group in 1996 [59]. The molybdenum complex **51** was immobilized onto silica at 70 °C. The loss of neopentane in **52** was postulated to be involved in the generation of an active silica-supported catalyst **53**. While the structure of **53** was inferred, the presence of a complex like **53** was confirmed by elemental analyses that yield a Mo/N/C ratio of 1/1/10 (Scheme 4). This immobilized complex **53** can prepare ROMP products of norbornene and *cis*-cyclooctene at 25 °C in high yield (74% and 85%, respectively). These ROMP products had *M*_n_ values of *ca.* 310,000 Da with a PDI of *ca*. 1.8. The metal analysis of molybdenum in ROMP products was not described.

**Scheme 5:**
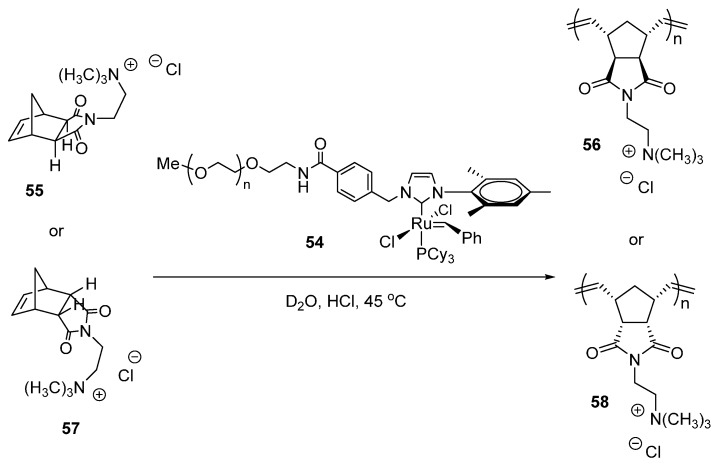
ROMP Reactions of **55** and **57** Catalyzed by **54**.

In attempting to develop a greener metathesis reaction process, the Grubbs group reported several syntheses of highly active PEG-bound ruthenium complexes that can be used in aqueous media to catalyze ROMP reactions. One example is the PEG_5000_ conjugated *N*-heterocyclic carbene-containing ruthenium benzylidene catalyst **54** [60] (Scheme 5). This catalyst initiated the ROMP of **55** to give polynorbornene **56** in 73% conversion after 24 h, as measured by ^1^H NMR spectroscopy in D_2_O. The rate of ROMP reaction with a catalyst like **55** depends on the rate of phosphine dissociation. In this case the dissociation of phosphine from catalyst **54** may be disfavored in water. However, the protonation of free phosphine by HCl could in principle inhibit re-association of the phosphine ligand. In this case, the rate of polymerization was dramatically increased to 95% conversion in 15 min when 1 equiv of HCl, relative to catalyst **54**, was present in the reaction. This chemistry is similar to the Phase Transfer Activation (PTA) principle used by the Gladysz and Bazzi groups where a phosphine that is more soluble in a fluorous or aqueous phase is used with a Grubbs catalyst under biphasic conditions where the phosphine separation into a fluorous or aqueous phase precludes re-association [61,62]. In the Grubbs’ work, the PEG-bound ruthenium catalyst **54** was shown to catalyze a ROMP reaction of the monomer *endo*- **57** with 86% conversion to polymer **58** within 14 h. It is known that this *endo*-monomer is a more challenging ROMP substrate than the *exo*-monomer **55** [60]. The extent to which the ROMP products were contaminated by residues of the PEG-bound ruthenium complex **54** was not reported.

**Scheme 6:**
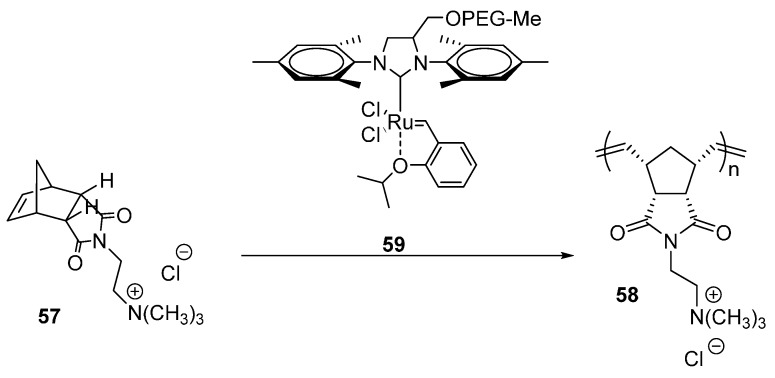
ROMP Reaction of **57** Catalyzed by PEG-Supported Hoveyda-Grubbs Catalyst **59**.

The complex **59** prepared with a PEG_2000_ support that was used in RCM and CM reactions of substrates like 2-allyl-*N*,*N*,*N*-trimethylpent-4-en-1-aminium chloride and allyl alcohol in water has also been used in the ROMP reaction of **57** (Scheme 6) [63]. The polymerization of **57** occurred in 100% conversion in 5 h. The removal process of the PEG-bound ruthenium complex **59** from the products **58** was not discussed at least in terms of the catalyst and products in a ROMP reaction.

**Scheme 7:**
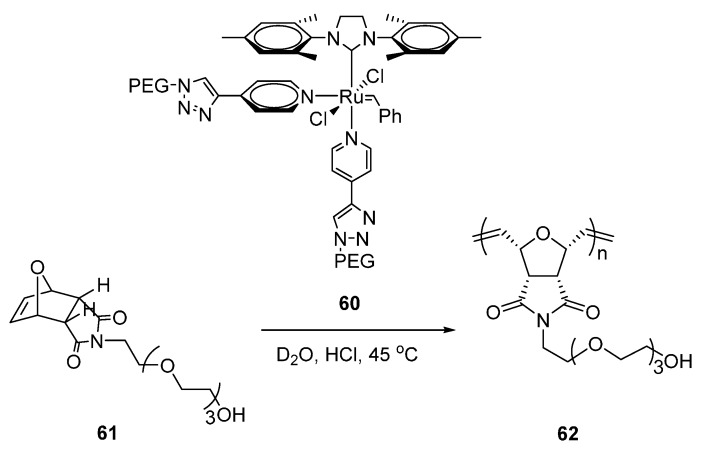
ROMP Reaction of **61** Catalyzed by PEG-Supported Hoveyda-Grubbs Catalyst **60**.

The immobilization of a highly active Grubbs 3rd generation catalyst using a PEG_350_ support has also been reported [64]. Emrick and co-workers synthesized a PEG-supported Grubbs 3rd generation by ligand exchange between pyridine ligands and PEG-bound pyridine ligands. This PEG-supported catalyst **60** was used to catalyze ROMP reactions in water (Scheme 7). The results showed that this catalyst works well at pH 1.5 with quantitative conversion of monomer **61** to polymer **62** in 2 h at room temperature. It was noted that the activity of **60** decreased with an increasing in pH, e.g., 23% conversion at pH 7 in 2 h at room temperature. However, with an addition of a Brønsted acid, *i.e.*, CuSO_4_ and CuBr_2_, the conversion of monomer **61** was improved from 23% to 70%. The PDI of the polymer products prepared from **60** ranged from 1.3 to 1.5. The amounts of Ru content in the products were not reported.

**Figure 11:**
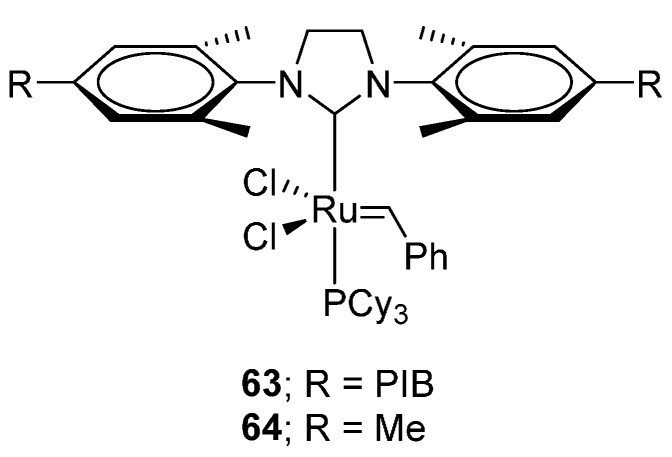
Polyisobutylene (PIB_1000_)-supported Grubbs second generation catalysts **63** and Grubbs catalyst **64**.

**Scheme 8:**
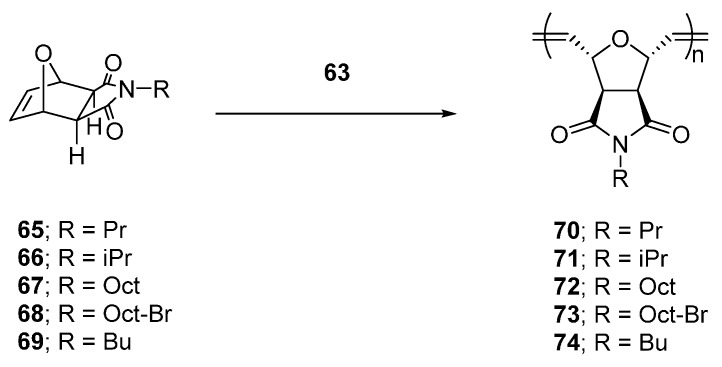
ROMP Reactions of **65**–**69** Catalyzed by **63**.

An early report of the use of a PIB-supported catalyst was part of a study of a polyisobutylbenzylidene-supported Ru catalyst that was used in ring closing metathesis. That work showed that this “boomerang” catalyst could be used in ROMP with *ca.* 3% leaching of the charged Ru [50]. Subsequently in 2012, a more effective Ru separation was achieved by the use of PIB-supported catalyst **45** in ROMP of norbornene derivatives as described in a report by Bazzi and Bergbreiter [65]. The catalyst **45** can be separated from the polymer product at the end of the reaction by dissolving the dry crude mixture of polymer and PIB-supported catalyst in an equi-volume mixture of DMF and heptane. The catalyst-containing heptane layer was then removed and the DMF layer was then evaporated to dryness to afford the crude polymer as an off-white solid. The crude polymer was dissolved in dichloromethane (DCM) and precipitated into an excess amount of hexane to afford the polymer product ready for analysis for properties and ruthenium contamination level. Polymers prepared from catalyst **45** have a nearly identical *E*/*Z* ratio to those prepared by their low molecular weight counterparts with PDI values ranging from 1.39 to 1.78. The amounts of ruthenium content in the resulting polymers were 111–228 ppm. In subsequent work by Bazzi and Bergbreiter, the activities and recoverability of a PIB-supported Grubbs second generation catalyst **63** formed from PIB with an *M*_n_ of 1000 Da (Figure 11) in ROMP was studied [66] in a polymerization of norbornene (Scheme 8). The polymerization rate of norbornene using **63** was 93% which is similar to that of its low molecular weight counterpart **64** (99% conversion, after 60 min). However, complex **63** exhibited a faster ROMP initiation with 70% conversion in 10 min than that of **64** with 30% conversion at the same time period. The PDI values of the polymer products **70**–**74** analyzed by GPC ranged from 1.11 to 2.34. The removal of catalyst **63** was examined in the ROMP reactions of monomer **65**–**69**. The process used in separation of residues from catalyst **63** and the polymer product involved dissolving the dry crude mixture of polymer product and Ru catalyst residue in a minimum amount of DCM and precipitating the mixture in hexane affording the homopolymer as a white solid. The Ru contamination level of polymers **70**–**74** prepared by **63** varied from 71 to 252 ppm.

**Figure 12:**
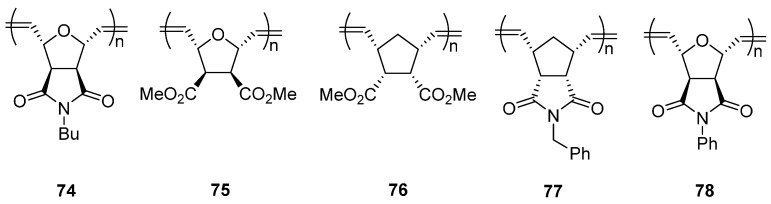
ROMP products **74**–**78**.

The PE_Olig_-supported ruthenium catalyst **46** has also been used in ROMP reactions of norbornene derivatives [67]. Examples of polymer prepared using **46** are shown in Figure 12. This PE_Olig_-supported catalyst **60** was used to prepare polymers **74**–**78** using the same solid/liquid separation concept as was used previously in reactions where this catalyst was used to prepare RCM products. In this case, separation of the ROMP products from **46** was performed by filtration of reaction mixture through Celite and 0.2 μm filter to yield colorless solution containing polymer products. The resulting solution was concentrated and then precipitated in hexane to isolate polymers **74**–**78**. In reactions with catalyst **46**, it was found that the prior scheme where polyethylene oligomers were shown to be useful cosolvents facilitate Ru residue separations in ROMP chemistry [67]. Adding unfunctionalized polyethylene oligomers as a cosolvent further facilitated separations of the product polymer and catalyst residues. For example, when a commercially available unfunctionalized polyethylene (Polywax) (*M*_n_ = 400) [68] was added as a co-solvent to PE_Olig_-supported catalyst, the leaching of Ru residues from catalyst **60** decreased to 19–26 ppm range (*ca*. 0.5% of the charged Ru catalyst). In addition, control experiments showed that heating a suspension of **46** and Polywax to form a solution followed by cooling and filtration led to only 0.04% leaching of Ru leaching, a value that increased to 0.42% (0.84 ppm, amount of Ru in the solution) if the heated solution of **46**, Polywax, and THF was quenched with butyl vinyl ether. This suggests that most of the *ca.* 19–26 ppm Ru residues observed in the ROMP products may be due to some side reactions that occur during the quenching of the living polymer and not from the polymerization process involving **46**. The resulting polymers **74**–**78** have the same *E*/*Z* ratio as those to those prepared by its low molecular weight counterpart **2**, comparable *M*_n_ values, and PDIs that ranged from 1.23 to 1.84.

## 5. Conclusions

Although many examples of newer versions of olefin metathesis catalysts with improved reactivity, air and moisture stability, functional groups tolerance, and stereoselectivity have been reported since the discovery of the first metathesis catalysts, challenges still remain. These challenges include: (i) the high cost of the transition metal complexes used as catalysts or precatalysts; (ii) the cost and/or tediousness of the ligand syntheses; (iii) the potential environmental toxicological or practical concerns that ensue when there is significant metal or ligand contamination in the product; and (iv) the cost of and waste generated when metal or ligand contaminates have to be removed by post reaction processing steps. Several approaches have been proposed to address these issues. One approach is to reduce the amount of catalyst used in the reaction. Lowering the wt % of catalyst will necessarily reduce catalyst cost, ligand cost, and product contamination. However, examples of this strategy can be limited, especially in polymerizations where each polymer chain has one catalyst site. An alternative strategy that has proven to be more versatile is to immobilize the catalyst on either heterogeneous or homogeneous supports. Such scheme allow the supported catalysts to be recovered and subsequently reused or, in other cases, effect separation of inactive catalyst residues from products. Several examples of this chemistry have been reported in the past two decades showing the success in using this strategy to address the issues mentioned earlier, especially those focused on the removal of metal contamination in the final products. The benefit of using supported olefin metathesis catalysts to effectively minimize the level of metal contamination in the products is significantly increased in the case of ROMP reactions when reducing the amount of catalyst might not be an option. Although there is still room for further development (such as recoverability and application in asymmetric synthesis) before the strategy of catalyst immobilization can be widely used in both industry and academia, its potential to be recognized as a standard tool in synthetic applications is not to be underestimated.

## References

[1] Grubbs R.H. (2003). Handbook of Metathesis.

[2] Grela K. (2014). Olefin Metathesis: Theory and Practice.

[3] Grubbs R.H. (2006). Olefin-metathesis catalysts for the preparation of molecules and materials. Angew. Chem. Int. Ed..

[4] Schrock R.R. (2006). Multiple metal–carbon bonds for catalytic metathesis reactions. Angew. Chem. Int. Ed..

[5] Vougioukalakis G.C. (2012). Removing ruthenium residues from olefin metathesis reaction products. Chem. Eur. J..

[6] Chu D.S.H., Schellinger J.G., Shi J., Convertine A.J., Stayton P.S., Pun S.H. (2012). Application of living free radical polymerization for nucleic acid delivery. Acc. Chem. Res..

[7] Kuhn K.M., Bourg J.-B., Chung C.K., Virgil S.C., Grubbs R.H. (2009). Effects of NHC-backbone substitution on efficiency in ruthenium-based olefin metathesis. J. Am. Chem. Soc..

[8] Hong S.H., Day M.W., Grubbs R.H. (2004). Decomposition of a key intermediate in ruthenium-catalyzed olefin metathesis reactions. J. Am. Chem. Soc..

[9] Courchay F.C., Sworen J.C., Ghiviriga I., Abboud K.A., Wagener K.B. (2006). Understanding structural isomerization during ruthenium-catalyzed olefin metathesis: A deuterium labeling study. Organometallics.

[10] Dinger M.B., Mol J.C. (2002). High turnover numbers with ruthenium-based metathesis catalysts. Adv. Synth. Catal..

[11] Merrifield R.B. (1963). Solid phase peptide synthesis. I. The Synthesis of a tetrapeptide. J. Am. Chem. Soc..

[12] Collman J.P., Belmont J.A., Brauman J.I. (1983). A silica-supported rhodium hydroformylation catalyst: Evidence for dinuclear elimination. J. Am. Chem. Soc..

[13] Drago R.S., Pribich D.C. (1985). A new method for enhancing site isolation on silica gel and for improving the lifetime of site-isolated catalysts. Inorg. Chem..

[14] Töllner K., Popovitz-Biro R., Lahav M., Milstein D. (1997). Impact of molecular order in Langmuir-Blodgett films on catalysis. Science.

[15] Annis D.A., Jacobsen E.N. (1999). Polymer-supported chiral Co(salen) complexes: Synthetic applications and mechanistic investigations in the hydrolytic kinetic resolution of terminal epoxides. J. Am. Chem. Soc..

[16] Nguyen S.T., Grubbs R.H. (1995). The syntheses and activities of polystyrene-supported olefin metathesis catalysts based on Cl_2_(PR_3_)_2_Ru=CH–CH=CPh_2_. J. Organomet. Chem..

[17] Ahmed M., Barrett A.G.M., Braddock D.C., Cramp S.M., Procopiou P.A. (1999). A recyclable “boomerang” polymer-supported ruthenium catalyst for olefin metathesis. Tetrahedron Lett..

[18] Schürer S.C., Gessler S., Buschmann N., Blechert S. (2000). Synthesis and application of a permanently immobilized olefin-metathesis catalyst. Angew. Chem. Int. Ed..

[19] Weskamp T., Kohl F.J., Hieringer W., Gleich D., Herrmann W.A. (1999). Highly active ruthenium catalysts for olefin metathesis: The synergy of *N*-heterocyclic carbenes and coordinatively labile ligands. Angew. Chem. Int. Ed..

[20] Randl S., Buschmann N., Connon S.J., Blechert S. (2001). Highly efficient and recyclable polymer-bound catalyst for olefin metathesis reactions. Synlett.

[21] Mennecke K., Grela K., Kunz U., Kirschning A. (2005). Immobilisation of the grubbs III olefin metathesis catalyst with polyvinyl pyridine (PVP). Synlett.

[22] Prühs S., Lehmann C.W., Fürstner A. (2004). Preparation, reactivity, and structural peculiarities of hydroxyalkyl-functionalized “second-generation” ruthenium carbene complexes. Organometallics.

[23] Allen D.P., van Wingerden M.M., Grubbs R.H. (2009). Well-defined silica-supported olefin metathesis catalysts. Org. Lett..

[24] Lim J., Seong Lee S., Ying J.Y. (2010). Mesoporous silica-supported catalysts for metathesis: Application to a circulating flow reactor. Chem. Commun..

[25] Bek D., Gawin R., Grela K., Balcar H. (2012). Ruthenium metathesis catalyst bearing chelating carboxylate ligand immobilized on mesoporous molecular sieve SBA-15. Catal. Commun..

[26] Monge-Marcet A., Pleixats R., Cattoën X., Man M.W.C. (2013). Catalytic applications of recyclable silica immobilized NHC–ruthenium complexes. Tetrahedron.

[27] Blanc F., Copéret C., Thivolle-Cazat J., Basset J.-M., Lesage A., Emsley L., Sinha A., Schrock R.R. (2006). Surface *versus* molecular siloxy ligands in well-defined olefin metathesis catalysts: [{(RO)_3_SiO}Mo(=NAr)(=CH*t*Bu)(CH_2_*t*Bu)]. Angew. Chem. Int. Ed..

[28] Lopez L.P.H., Schrock R.R. (2004). Formation of dimers that contain unbridged W(IV)/W(IV) double bonds. J. Am. Chem. Soc..

[29] Blanc F., Thivolle-Cazat J., Basset J.-M., Copéret C., Hock A.S., Tonzetich Z.J., Schrock R.R. (2007). Highly active, stable, and selective well-defined silica supported Mo imido olefin metathesis catalysts. J. Am. Chem. Soc..

[30] Blanc F., Rendon N., Berthoud R., Basset J.-M., Coperet C., Tonzetich Z.J., Schrock R.R. (2008). Dramatic enhancement of the alkene metathesis activity of Mo imido alkylidene complexes upon replacement of one tBuO by a surface siloxy ligand. Dalton Trans..

[31] Hultzsch K.C., Jernelius J.A., Hoveyda A.H., Schrock R.R. (2002). The first polymer-supported and recyclable chiral catalyst for enantioselective olefin metathesis. Angew. Chem. Int. Ed..

[32] Dolman S.J., Hultzsch K.C., Pezet F., Teng X., Hoveyda A.H., Schrock R.R. (2004). Supported chiral Mo-based complexes as efficient catalysts for enantioselective olefin metathesis. J. Am. Chem. Soc..

[33] Mayr M., Wang D., Kröll R., Schuler N., Prühs S., Fürstner A., Buchmeiser M.R. (2005). Monolithic disk-supported metathesis catalysts for use in combinatorial chemistry. Adv. Synth. Catal..

[34] Dupont J., de Souza R.F., Suarez P.A.Z. (2002). Ionic liquid (molten salt) phase organometallic catalysis. Chem. Rev..

[35] Buijsman R.C., van Vuuren E., Sterrenburg J.G. (2001). Ruthenium-catalyzed olefin metathesis in ionic liquids. Org. Lett..

[36] Semeril D., Olivier-Bourbigou H., Bruneau C., Dixneuf P.H. (2002). Alkene metathesis catalysis in ionic liquids with ruthenium allenylidene salts. Chem. Commun..

[37] Audic N., Clavier H., Mauduit M., Guillemin J.-C. (2003). An Ionic liquid-supported ruthenium carbene complex: A robust and recyclable catalyst for ring-closing olefin metathesis in ionic liquids. J. Am. Chem. Soc..

[38] Yao Q., Sheets M. (2005). An ionic liquid-tagged second generation Hoveyda–Grubbs ruthenium carbene complex as highly reactive and recyclable catalyst for ring-closing metathesis of di-, tri- and tetrasubstituted dienes. J. Organomet. Chem..

[39] Thurier C., Fischmeister C., Bruneau C., Olivier-Bourbigou H., Dixneuf P.H. (2007). Ionic imidazolium containing ruthenium complexes and olefin metathesis in ionic liquids. J. Mol. Catal. A Chem..

[40] Fustero S., Simón-Fuentes A., Barrio P., Haufe G. (2015). Olefin metathesis reactions with fluorinated substrates, catalysts, and solvents. Chem. Rev..

[41] Horváth I.T., Rábai J. (1994). Facile catalyst separation without water: Fluorous biphase hydroformylation of olefins. Science.

[42] Yao Q., Zhang Y. (2004). Poly(fluoroalkyl acrylate)-bound ruthenium carbene complex: A fluorous and recyclable catalyst for ring-closing olefin metathesis. J. Am. Chem. Soc..

[43] Matsugi M., Curran D.P. (2005). Synthesis, Reaction, and recycle of light fluorous grubbs−hoveyda catalysts for alkene metathesis. J. Org. Chem..

[44] Matsugi M., Kobayashi Y., Suzumura N., Tsuchiya Y., Shioiri T. (2010). Synthesis and RCM reactions using a recyclable grubbs−hoveyda metathesis catalyst activated by a light fluorous tag. J. Org. Chem..

[45] Bergbreiter D.E. (2002). Using soluble polymers to recover catalysts and ligands. Chem. Rev..

[46] Bergbreiter D.E., Tian J., Hongfa C. (2009). Using soluble polymer supports to facilitate homogeneous catalysis. Chem. Rev..

[47] Gravert D.J., Janda K.D. (1997). Organic synthesis on soluble polymer supports: Liquid-phase methodologies. Chem. Rev..

[48] Varray S., Lazaro R., Martinez J., Lamaty F. (2003). New soluble-polymer bound ruthenium carbene catalysts: Synthesis, characterization, and application to ring-closing metathesis. Organometallics.

[49] Yao Q. (2000). A Soluble polymer-bound ruthenium carbene complex: A robust and reusable catalyst for ring-closing olefin metathesis. Angew. Chem. Int. Ed..

[50] Hongfa C., Tian J., Bazzi H.S., Bergbreiter D.E. (2007). Heptane-soluble ring-closing metathesis catalysts. Org. Lett..

[51] Hongfa C., Su H.-L., Bazzi H.S., Bergbreiter D.E. (2009). Polyisobutylene-anchored *N*-heterocyclic carbene ligands. Org. Lett..

[52] Hobbs C., Yang Y.-C., Ling J., Nicola S., Su H.-L., Bazzi H.S., Bergbreiter D.E. (2011). Thermomorphic polyethylene-supported olefin metathesis catalysts. Org. Lett..

[53] Hansen L.C., Sievers R.E. (1974). Highly permeable open-pore polyurethane columns for liquid chromatography. J. Chromatogr. A.

[54] Hjertén S., Liao J.-L., Zhang R. (1989). High-performance liquid chromatography on continuous polymer beds. J. Chromatogr. A.

[55] Hjerten S., Yi-Ming Li Y., Liao J.-L., Mohammad J., Nakazato K.I., Pettersson G. (1992). Continuous beds: High-resolving, cost-effective chromatographic matrices. Nature.

[56] Tripp J.A., Stein J.A., Svec F., Fréchet J.M.J. (2000). “Reactive filtration”: Use of functionalized porous polymer monoliths as scavengers in solution-phase synthesis. Org. Lett..

[57] Tripp J.A., Svec F., Fréchet J.M.J. (2001). Grafted macroporous polymer monolithic disks: A new format of scavengers for solution-phase combinatorial chemistry. J. Comb. Chem..

[58] Mayr M., Mayr B., Buchmeiser M.R. (2001). Monolithic materials: New high-performance supports for permanently immobilized metathesis catalysts. Angew. Chem. Int. Ed..

[59] Herrmann W.A., Stumpf A.W., Priermeier T., Bogdanović S., Dufaud V., Basset J.-M. (1996). A Molecularly defined, grafted olefin metathesis catalyst from tris(neopentyl)-nitridomolybdenum(VI). Angew. Chem. Int. Ed..

[60] Gallivan J.P., Jordan J.P., Grubbs R.H. (2005). A neutral, water-soluble olefin metathesis catalyst based on an *N*-heterocyclic carbene ligand. Tetrahedron Lett..

[61] Da Costa R.C., Gladysz J.A. (2007). Syntheses and reactivity of analogues of grubbs’ second generation metathesis catalyst with fluorous phosphines: A new phase-transfer strategy for catalyst activation. Adv. Synth. Catal..

[62] Tuba R., Corrêa da Costa R., Bazzi H.S., Gladysz J.A. (2012). Phase transfer activation of fluorous analogs of grubbs’ second-generation catalyst: Ring-opening metathesis polymerization. ACS Catal..

[63] Hong S.H., Grubbs R.H. (2006). Highly active water-soluble olefin metathesis catalyst. J. Am. Chem. Soc..

[64] Samanta D., Kratz K., Zhang X., Emrick T. (2008). A synthesis of PEG- and phosphorylcholine-substituted pyridines to afford water-soluble ruthenium benzylidene metathesis catalysts. Macromolecules.

[65] Al-Hashimi M., Hongfa C., George B., Bazzi H.S., Bergbreiter D.E. (2012). A phase-separable second-generation hoveyda-grubbs catalyst for ring-opening metathesis polymerization. J. Polym. Sci. A Polym. Chem..

[66] Al-Hashimi M., Bakar M.D.A., Elsaid K., Bergbreiter D.E., Bazzi H.S. (2014). Ring-opening metathesis polymerization using polyisobutylene supported Grubbs second-generation catalyst. RSC Adv..

[67] Suriboot J., Hobbs C.E., Guzman W., Bazzi H.S., Bergbreiter D.E. (2015). Polyethylene as a cosolvent and catalyst support in ring-opening metathesis polymerization. Macromolecules.

[68] Baker Hughes Polywax. http://www.bakerhughes.com/news-and-media/resources/technical-data-sheet/polywax-polyethylenes.

